# Biological and computational assessments of thiazole derivative-reinforced bile salt enriched nano carriers: a new gate in targeting SARS-CoV-2 spike protein†

**DOI:** 10.1039/d4ra07316a

**Published:** 2024-12-09

**Authors:** Mohamed Y. Zakaria, Ayman Abo Elmaaty, Rabeh El-Shesheny, Radwan Alnajjar, Omnia Kutkat, Sana Ben Moussa, Abdullah Yahya Abdullah Alzahrani, Sally A. El-Zahaby, Ahmed A. Al-Karmalawy

**Affiliations:** a Department of Pharmaceutics and Industrial Pharmacy, Faculty of Pharmacy, King Salman International University Ras Sudr 46612 South Sinai Egypt mohamed.yehia@ksiu.edu.eg; b Department of Pharmaceutics and Industrial Pharmacy, Faculty of Pharmacy, Port Said University Port Said 42526 Egypt; c Medicinal Chemistry Department, Faculty of Pharmacy, Port Said University Port Said 42526 Egypt; d Medicinal Chemistry Department, Clinical Pharmacy Program, East Port Said National University Port Said 42526 Egypt; e Center of Scientific Excellence for Influenza Viruses, Water Pollution Research Department, Environmental Research Institute, National Research Centre Dokki-Giza 12622 Egypt; f CADD Unit, Faculty of Pharmacy, Libyan International Medical University Benghazi 16063 Libya; g Department of Chemistry, Faculty of Science, University of Benghazi Benghazi 16063 Libya; h Department of Chemistry, Faculty of Science and Arts, King Khalid University Mohail Assir Saudi Arabia; i Department of Pharmaceutics and Industrial Pharmacy, PharmD Program, Egypt-Japan University of Science and Technology (E-Just) Alexandria Egypt; j Department of Pharmaceutical Chemistry, College of Pharmacy, The University of Mashreq Baghdad 10023 Iraq; k Department of Pharmaceutical Chemistry, Faculty of Pharmacy, Horus University-Egypt New Damietta 34518 Egypt akarmalawy@horus.edu.eg

## Abstract

There is merit in investigating novel therapeutic molecules that hit vital targets during the viral infection cycle *i.e.* disrupting the interaction between SARS-CoV-2's spike glycoprotein and the host's angiotensin converting enzyme 2 (ACE2) receptor, potentially offering new avenues for treatment. Accordingly, lipid-based vesicular systems like liposomes or niosomes are frequently utilized to overcome these hurdles. Thus, chemically synthesized compounds were encapsulated within PEGylated bilosomes (PBs) to improve their solubility and intestinal permeability, thereby enhancing their anti-SARS-CoV-2 effectiveness. The formulae were prepared according to 2^3^ full factorial design which was also used to explore the impact of the change in predetermined formulation variables on the properties of the prepared vesicles (entrapment efficiency EE%, particle size PS, and zeta potential ZP). Additionally, the optimized formula (F4) which is composed of 3% bile salt (BS), 40 mg 1,2-distearoyl-*sn*-glycero-3-phosphoethanolamine-*N*-[amino(polyethylene glycol)-2000] (DSPE) and sodium deoxycholate (SDC) as a bile salt, was selected as an optimum formula with desirability value 0.674 using Design Expert® software. Both the *in vitro* release and *ex vivo* experiments results confirmed the significant superiority of the F4 over the drug dispersion. Both cytotoxicity and anti-SARS-CoV-2 activity of all examined compound-loaded PBs (PB3a–PB3g) were assessed in Vero E6 cells *via* MTT assay. Both compounds PB3c and PB3g displayed the highest IC_50_ values (0.71 and 1.25 μg mL^−1^, respectively) ensuring their superior antiviral potential. Moreover, it was revealed that PB3c demonstrated more than 80% virucidal activity and over 80% inhibition of viral adsorption with little effect on the viral replication ∼(5–10%). Moreover, molecular docking and dynamic studies were conducted to pursue the binding affinities of the investigated compounds towards the ACE2 target of the SARS-CoV-2 spike protein, assuring their feasible inhibitory potential. Collectively, the investigated compound-loaded PBs can be treated as promising lead drug delivery panels for COVID-19 management.

## Abstract 2

Thiazole derivative-reinforced bile salt enriched nano carriers: a new frontier in targeting SARS-CoV-2 spike protein.

## Graphical abstract

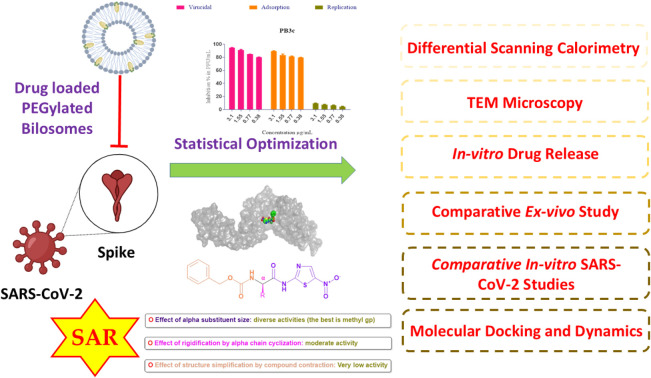

## 1 Introduction

In December 2019, a new coronavirus, COVID-19, was initially detected in China. The virus outbreak originated in Wuhan city and rapidly spread globally. With the severity of the virus and its widespread impact, the World Health Organization (WHO) officially declared COVID-19 as a pandemic on March 11, 2020.^1^ The virus has had a detrimental impact on the global economy and social life, leading to widespread disruption, lockdowns, and changes in everyday activities to curb its spread.^2^ Preventive measures, such as vaccination campaigns, social distancing, and mask-wearing, have been implemented to mitigate its effects and protect vulnerable populations.

During the early stages of viral infection, individuals often experience mild symptoms, which commonly include fever, muscle pain (myalgia), and a dry cough.^3^ As the disease progresses to more advanced stages, pulmonary symptoms become prominent, with individuals developing difficulty in breathing (dyspnea) and experiencing low levels of oxygen in the blood (hypoxia). It is important to monitor these symptoms closely and seek medical attention promptly, especially in vulnerable individuals, to ensure appropriate care and management of the disease.^3^ Indeed, even as the viral load typically decreases during the course of the infection, some patients may experience a worsening of their condition. This deterioration is often attributed to an uncontrolled systemic inflammatory response, commonly referred to as a cytokine storm. The cytokine storm is an excessive and dysregulated release of immune system signaling molecules, leading to severe inflammation. This inflammatory response can cause significant damage to lung tissues and other organs.^4^

Indeed, SARS-CoV-2, like SARS-CoV and MERS-CoV, belongs to the genus β-coronavirus and shares certain structural characteristics. It is an enveloped virus with a positive-stranded RNA genome.^5^ SARS-CoV-2 is mainly composed of four essential structural proteins. These structural proteins are; envelope protein (it plays a vital role in the assembly and release of new virus particles), membrane protein (this protein is integral to the viral envelope and is involved in virus assembly and budding), spike protein (the spike protein is responsible for binding to the host cell receptor (angiotensin-converting enzyme 2, or ACE2) and mediating viral entry into the host cells), and nucleocapsid protein (this protein encapsulates the viral RNA, forming the nucleocapsid, which is crucial for viral replication and assembly).^5^

**Fig. 1:**
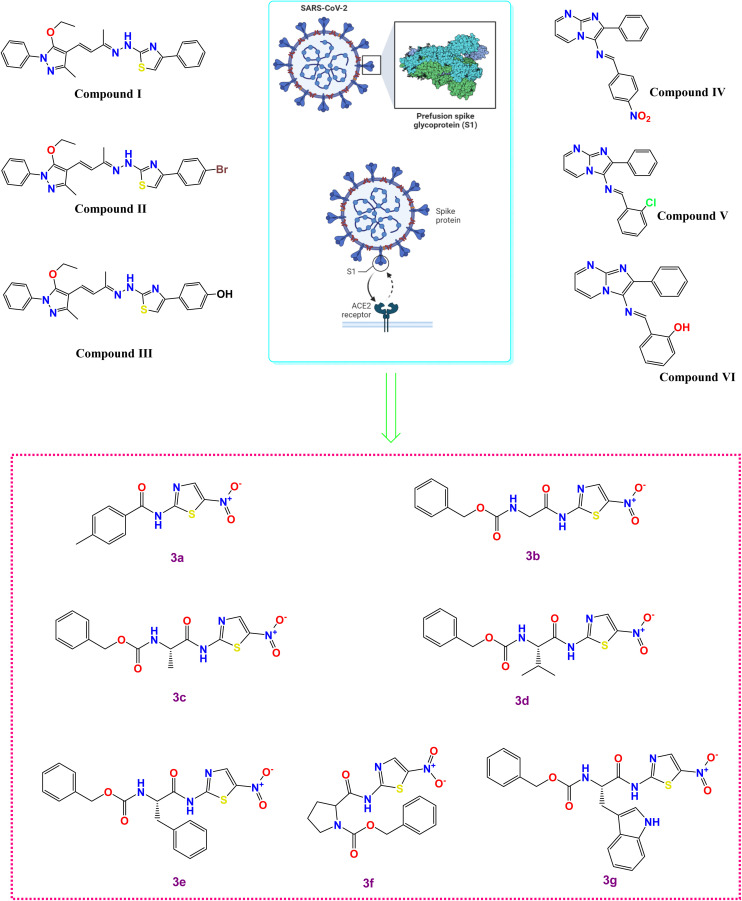
Some reported SARS-CoV-2 spike inhibitors illustrating spike composition and its interaction with ACE2 for viral entry along with our investigated compounds (3a–g).

Exactly, the entry of SARS-CoV-2 into host cells is a multi-step process that involves the spike (S) glycoprotein on the virus's surface and the ACE2 receptors on the cell surface. The initial step is the binding of the S glycoprotein to the ACE2 receptors, which serves as the first interaction between the virus and the host cell, Fig. 1.^6^ Following the S glycoprotein-ACE2 receptor binding, conformational changes occur in the S protein. These changes facilitate the fusion of the viral envelope with the cell membrane, enabling the virus to enter the host cell. This fusion process occurs through an endosomal pathway, where the viral envelope and the cell membrane merge, leading to the release of the virus's genetic material (RNA) into the host cell's cytoplasm.^6^ Hence, understanding the mechanism of viral entry is essential for developing potential therapies and vaccines that can target these interactions and prevent viral infection.

Furthermore, while the development and distribution of COVID-19 vaccines have been significant achievements in combating the pandemic, no vaccine is 100% effective, and breakthrough infections can occur, especially with the emergence of new variants.^6^ Besides, although vaccines play a crucial role in reducing the severity of the disease and preventing hospitalizations and deaths, they may not completely prevent transmission. Moreover, the global distribution and administration of vaccines have been unequal, with underdeveloped countries facing challenges in obtaining sufficient vaccine supplies and ensuring equitable access to vaccination. This has resulted in slower vaccination rates and potential gaps in protection against the virus in some regions.^6^

Given these factors, the demand for effective pharmacological treatments for COVID-19 remains urgent. Such treatments could provide an additional layer of protection, especially for vulnerable populations or individuals for whom vaccines may not be fully effective.^7^ Ongoing research efforts are focused on developing antiviral drugs and other therapies to treat COVID-19 and reduce its severity and impact on public health worldwide. On the other hand, The S glycoprotein constitutes one of the SARS-CoV-2 primary structural proteins. This protein is situated on the virus's surface and plays a crucial role in facilitating viral entry into human host cells. It accomplishes this by binding to the ACE2 receptor. Exploring ways to interfere with the interactions between the S glycoprotein and ACE2 receptor may open up novel therapeutic approaches.^8^

The literature revealed that different chemical compounds were designed and synthesized as potent inhibitors of SARS-CoV-2 entry into the host cells. Pyrazolone-based compounds were synthesized as potent anti-spike agents (compounds I–III, Fig. 1).^9^ Besides, novel imidazo[1,2-*a*]pyrimidine derivatives were designed and synthesized as potential dual inhibitors of *h*ACE2 and spike protein for blocking SARS-CoV-2 cell entry (compounds IV–VI, Fig. 1).^10^

Although various inhibitors displayed strong inhibitory effects against SARS-CoV-2, however, most of these drugs were halted during preclinical testing and terminated during *in vivo* studies due to concerns related to pharmacodynamics or pharmacokinetics.^11^ One of the most common reasons for the low absorption and limited oral bioavailability of candidate drugs is their reduced intestinal permeability, coupled with their susceptibility to extensive metabolism in the gastrointestinal tract (GIT) and liver.^12^ Thus, a lipid-based vesicular system, such as liposomes or niosomes, is commonly employed to overcome these challenges.^13^ Emulsomes are a type of vesicular system that combine the features of lipidic bilayered vesicles and nanoemulsions, offering the advantages of both.^14^ Emulsomes consist primarily of two essential components: a solid lipid core surrounded by a phospholipid outer layer, enhancing the stability of the vesicle. These emulsomes effectively accommodate both hydrophilic and lipophilic drugs, while also addressing the limitations of conventional vesicular systems, such as the tendency to aggregate and a higher drug leakage rate.^15^ Furthermore, surface PEGylation of emulsomes can enhance vesicle stability and extend their presence in the systemic circulation.^16^ Consequently, emulsomes are considered a promising carrier for candidate drugs, enhancing the bioavailability and antiviral activity of these drugs.

As a prosperous means for oral delivery of therapeutic moieties, vesicular systems such as liposomes, emulsomes and niosomes could be proposed. This could be owing to their flexible structure and versatility, unfortunately, there are many drawbacks associated with the utilization of the traditional vesicular systems as their liability limited stability, encapsulation, and scaling-up problems. Thus, bilosomes (BS) were proposed as a fruitful system to overcome those problems^17^ and the vesicular deformability and elasticity acquired due to the presence of BS.^18^ Furthermore, the packing of the vesicular structure of BS is preserved in spite of the harsh conditions in GIT as acidic pH and digestive enzymes which then prohibit the rapid, dumped drug release and keep the drug intact and undecomposed.^19^ Additionally, the utilization of a PEGylated edge activator will aid in enhancing the vesicles' stability by keeping the vesicles apart from each other, releasing the drug in a more controlled manner, and prolonging the drug circulation in the blood *via* prohibition of the attachment of the vesicles to plasma proteins.^20,21^

Accordingly, the synthetic drugs were loaded on PEGylated bilosomes (PBs) aspiring to enhance their solubility and intestinal permeability besides boosting their anti-SARS-CoV-2 activity. The formulae were prepared using the solvent evaporation method and then they were *in vitro* characterized for determination of EE%, PS, and ZP. Additionally, the optimized formula was selected and involved in various characterizations: transmission electron microscopy (TEM), differential scanning calorimetry (DSC), comparative drug release, and *ex vivo* permeation study.

Finally, the promoted antiviral activity of the drug-loaded PBs was assessed on SARS-CoV-2 compared with the free unformulated drugs, and the mechanism by which the drug-loaded optimized formula was elucidated *via* mechanistic study.

## 2 Materials and Methods

### 2.1. Material

Span 60 and 1,2-distearoyl-*sn*-glycero-3-phosphoethanolamine-*N*-[amino(polyethylene glycol)-2000] (DSPE) were purchased from Sigma-Aldrich, Inc., St. Louis, MO, USA. DSPE was obtained from BASF Co. (Florham Park, NJ, USA). The sodium chloride, potassium dihydrogen orthophosphate, methanol, sodium hydroxide, magnesium chloride, chloroform, and absolute ethanol were purchased from El-Gomhouria Chemical Co., Cairo, Egypt. The dialysis membranes (Spectra/Pore®, cut-off 12 000–14 000) were purchased from Spectrum Laboratories Inc., Rancho Dominguez, CA, USA. All chemicals and solvents were of analytical grade and were used as received.

### 2.2. Methods

#### 2.2.1. Formulation of 3c-loaded PBs

The drug-loaded PBs were prepared to adopt slightly modified thin film hydration.^22^ Precisely, 100 mg Span 60, 50 mg cholesterol, 20 mg drug “3c” and (20 mg or 40 mg) DSPE were dissolved in a rounded bottom flask in a 20 mL mixture of ethanol and chloroform (2 : 1). The flask was attached to a rotary evaporator (Rotavapor, Heidolph VV 2000; Heidolph Instruments, Kelheim, Germany) for 30 min at 60 °C under reduced pressure for attaining a dry film and ensuring the total eradication of the solvents. Then the dry film was hydrated using 10 mL phosphate-buffered saline pH 7.4 solution containing (1% or 3%) of (SDC or sodium taurocholate (STC)) as bile salt for 2 h. The obtained dispersion of PBs formulae was subjected to more reduction in PS using an ultrasonic bath sonicator (Ultra Sonicator, Model LC 60/H Elma, Germany) for 10 min. The composition of the prepared formulae and their relevant characterization are demonstrated in Table 1.

**Table 1:** 2^3^ full factorial experimental design; experimental runs, independent variables, and estimated responses of 3c-loaded PBs

Formula	*A* (BS concentration) (%w/v)	*B* (DSPE amount) (mg)	C (BS type)	*Y*_1_ (EE%)	*Y*_2_ (PS) (nm)	*Y*_3_ (ZP) (mV)	PDI
F1	1	20	SDC	93.4 ± 2.6	332.8 ± 18.7	−28.4 ± 2.6	0.09 ± 0.04
F2	3	20	SDC	88.1 ± 3.2	271.2 ± 10.6	−34.3 ± 1.1	0.22 ± 0.06
F3	1	40	SDC	83.7 ± 1.9	221.6 ± 20.7	−36.8 ± 1.9	0.29 ± 0.05
F4	3	40	SDC	80.1 ± 2.8	181.5 ± 7.2	−40.1 ± 3.4	0.07 ± 0.02
F5	1	20	STC	77.4 ± 1.4	421.2 ± 22.4	−41.9 ± 2.9	0.36 ± 0.08
F6	3	20	STC	70.1 ± 3.5	383.4 ± 32.1	−44.6 ± 4.9	0.21 ± 0.06
F7	1	40	STC	62.4 ± 1.1	362.8 ± 38.2	−48.7 ± 3.8	0.42 ± 0.09
F8	3	40	STC	57.3 ± 1.8	330.1 ± 21.8	−52.1 ± 4.5	0.31 ± 0.05

#### 2.2.2. Model optimization and *in vitro* characterization of drug “3c”-loaded PBs

##### 2.2.2.1. Assessment of entrapment efficiency percentage (EE%)

Indirect determination of entrapped% of the drug in the prepared vesicles (EE%) was performed according to Abdelbary *et al.*^23^ Simply, 1 mL of the crude formula was centrifugated at 21 000 rpm and 4 °C using a cooling centrifuge (3K30, Sigma, Germany) for 1 h for the separation of the free unentrapped drug from the entrapped. The sedimented pellets were then washed off two times with phosphate-buffered saline pH 7.4 to assure the removal of the free traces of the drug and recentrifuged for 30 min post each washing and the clear supernatant was harvested. The supernatants were analyzed at *λ*_max_ 254 nm spectrophotometrically (Shimadzu, model UV-1601 PC, Kyoto, Japan) for determination of the concentration of the unentrapped drug after being diluted with methanol.^24^

EE% was computed utilizing the following equation:
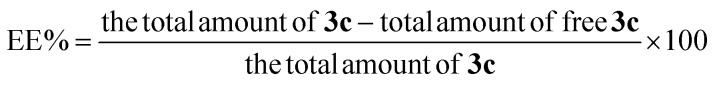


##### 2.2.2.2. Assessment of particle size (PS), polydispersity index (PDI) and zeta potential (ZP)

0.1 mL of the different formulae dispersion was diluted 10 times using distilled water for the investigation of PS, PDI (polydispersity index), and ZP for different bilosomal dispersions: 0.1 mL of formulae dispersions were 10 times diluted with distilled water. The diluted dispersion was analyzed using Malvern Zetasizer (Model ZEN3600, Malvern Instruments Ltd Worcestershire, UK).^25^

#### 2.2.3. Model optimization and choice of the optimized formula

The impact and significance of the formulation variables [BS concentration (%w/v) (*A*), DSPE amount (mg) (*B*), and BS type (*C*)] on the system responses (EE%, PS, PDI, and ZP) were analyzed using 23 full factorial design *via* Design-Expert® software version 13 (Stat-Ease, Inc., Minneapolis, Minnesota, USA). Moreover, the optimum formula exhibiting the highest desirability value (much closer to 1) was selected based on the following criteria: highest EE% and ZP (as absolute values and lowest PS and PDI) and then incorporated in various characterizations.^26^

#### 2.2.4. *In vitro* characterization of the optimized formula

##### 2.2.4.1. Differential scanning calorimetry (DSC)

The optimized formula dispersion was first lyophilized for solidification as follows: the dispersion was frozen (−20 °C), then the sample was transferred to a freeze-dryer (Novalyphe-NL 500 freeze-dryer, Savant Instruments, NY, USA) for lyophilization where it was kept at (−45 °C) for 24 h under reduced pressure.^27^ After that, for the assessment behaviors of pure drug, lyophilized 3c-loaded optimum formula and lyophilized blank optimum formula, a weight of 2 mg from each of them was added on an aluminum pan and subjected to a gradual increase in temperature reaching 400 °C in a nitrogen environment at a scanning rate of (10 °C min^−1^) using DSC (PerkinElmer, Waltham, MA).^28^

##### 2.2.4.2. TEM

The morphology of the optimized formula was determined *via* TEM (Joel JEM 2100, Tokyo, Japan), whereas, on a carbon grid with copper coating a droplet from the optimized formula was negatively stained by 1% phosphotungstic acid, then it was left to dry. Finally, the dried film was visualized.

##### 2.2.4.3. *In vitro* release experiment

In two separate glass cylinders, each 10 cm in length and 2.5 cm diameter, 1 mL of drug-loaded optimum formula after being diluted with 1 mL phosphate buffer (pH 7.4) was placed on the presoaked cellulose membrane which covered the end of the cylinders, while 1 mL of drug dispersion was placed in the other glass cylinder.^29^ Then the glass cylinders were connected to the shaft of the dissolution apparatus (Copley, DIS 8000, Nottingham, UK) which was allowed to revolve in 900 mL of phosphate buffer saline (pH 7.4) with an adjusted temperature of 37 ± 0.5 °C in the dissolution vessels.^30^ At different time intervals, 5 mL samples were taken from the dissolution medium which was replaced with fresh phosphate buffer to maintain the sink condition. The concentration of the drug in the samples was detected using a UV spectrophotometer at 254 nm.^31^

#### 2.2.5. Short-term stability study

The impact of storage on the optimal formula, after being preserved at 4 °C and room temperature for 3 months, was evaluated. At the end of the storage period, samples were collected and analyzed for EE%, PS, and ZP. These results were compared to the initial measurements taken before storage, with a change considered significant if *p* ≤ 0.05.^32,33^

#### 2.2.6. *Ex vivo* permeation study

The level of enhancement in the ability of the compound “3c” in permeation of intestinal mucosa from optimized formula compared to the drug suspension was investigated by adopting a non-everted rat intestinal sac model. This technique is very beneficial in analyzing the mechanism of drug absorption and taking into consideration the other physiological parameters that affect drug absorption as the presence of transporters and intestinal enzymes which can't be evaluated by the conduction of *in vitro* release experiments.

This method is considered a valuable method for investigating the *ex vivo* drug absorption mechanisms because of the involvement of transporters and intestinal enzymes in drug absorption and transport through the gut, which is not the case upon performing *in vitro* drug release studies.^30^ The small intestines were carefully washed with phosphate buffer saline (0.1 M, pH 7.4) after being separated from the animals, then they were segmented into 4 cm long pieces. 1 mL of the optimum formula and the drug suspension were placed in the intestinal segments then they were closed from both ends. The segments were finally placed in a continuously shaken beaker containing 20 mL of phosphate buffer saline (0.1 M, pH 7.4) in a shaking water bath and kept at 37 ± 0.5 °C. Throughout the 24 h of experiment duration, the samples were withdrawn and filtered using a syringe Millipore filter (0.4 μm), then the amount of the drug in the filtrate was estimated using HPLC at a *λ*_max_ of 254 nm. The apparent permeability coefficient (*P*_app_) was estimated *via* the equation adopted by Feng *et al.*^34^

### 2.3. *In vitro* studies

#### 2.3.1. MTT assay

The CC_50_ for each compound under investigation in VERO-E6 cells was determined using the 3-(4,5-dimethylthiazol-2-yl)-2,5-diphenyltetrazolium bromide (MTT) assay, following a previously described procedure.^35^ Minor modifications were made to the MTT assay method to calculate the minimum concentrations of the newly synthesized candidates that induce 50% toxicity in the cells (CC_50_). For a detailed explanation of the complete methodology, please refer to the ESI (S1 and S2).†

#### 2.3.2. Inhibitory concentration 50 (IC_50_)

The IC_50_ for each tested compound formula (PB3a–PB3g), representing the minimum concentration required to inhibit virus infectivity by 50% compared to the virus control, was determined.^36^ The detailed methodology for this calculation can be found in the ESI (S1 and S2).†

#### 2.3.3. Mode of action against SARS-CoV-2

The potential mode of action for the most potent formula (PB3c) concerning the inhibition of SARS-CoV-2 was investigated at three distinct stages of the virus propagation cycle, considering three main possible mechanisms:

(i) The formula's direct effect may involve inactivating the virus, leading to a reduction in virus viability (virucidal activity).

(ii) The formula's ability to hinder the virus's attachment to infected cells, blocking viral entry through membrane fusion (viral adsorption).

(iii) The formula's capability to inhibit viral budding and replication, thereby impeding the virus's ability to reproduce and spread.

Furthermore, the mentioned modes of action could contribute to the observed antiviral activities either independently or in combination. The interaction between PB3c and SARS-CoV-2 might be attributed to the three different modes of action described earlier. To investigate this, a plaque infectivity reduction assay was conducted following the procedure as reported in the ESI (S3).† This assay would help assess the effectiveness of PB3c in reducing the infectivity of the virus and provide insights into its antiviral mechanisms.

### 2.4. *In silico* studies

#### 2.4.1. Docking study

The seven examined candidates (3a, 3b, 3c, 3d, 3e, 3f, and 3g) were docked^37,38^ against the SARS-CoV-2 spike protein receptor binding domain to investigate their binding affinities. All members were sketched in ChemDraw and then prepared for docking by correcting their partial charges and minimizing their energy.^39,40^ Afterward, they were inserted in one database to be docked into the SARS-CoV-2 spike protein which was downloaded from the Protein Data Bank (PDB ID: 6XE1). The target SARS-CoV-2 spike protein was prepared before the docking step by correcting its missed parts, 3D hydrogenation of its atoms, and energy minimization as well.^41^ Finally, the docked compounds were ranked according to their binding scores and the superior candidates were selected for visualization and explanation.

#### 2.4.2. Molecular dynamics studies and MM-GBSA calculations

Desmond package (Schrödinger LLC)^42^ was used to apply the MD simulations.^43,44^ Moreover, the thermal_mmgbsa.py python script of Schrodinger was used to calculate the Molecular Mechanics Generalized Born Surface Area (MM-GBSA) energies for all complexes.^45,46^ The detailed MD methodology is represented in the ESI (S4 and S5).†

## 3 Results and discussion

### 3.1. Analysis of experimental design

The impact of the change in the selected independent variables: BS concentration (% w/v) (*A*), DSPE amount (mg) (*B*), and BS type (*C*) on the *in vitro* characteristics of the prepared PEGylated bilosmes adopting 2^3^ full factorials. The composition of the resulting eight experimental runs along with the results of their responses are demonstrated in Table 1. Furthermore, the values of both the predicted *R*^2^ and the adjusted *R*^2^ were deemed to be in harmony with each other as the difference between them in all responses did not exceed 0.2 as noticed in Table 2.^30^

**Table 2:** 2^3^ Output factorial analysis data of 3c-loaded BPs and the predicted responses, observed responses, and deviation percent of the optimum formula (F4)

Responses	EE (%)	PS (nm)	ZP (mV)
*R* ^2^	0.9852	0.9738	0.9928
Adjusted *R*^2^	0.9741	0.9541	0.9874
Predicted *R*^2^	0.9407	0.8951	0.9712
Adequate precision	25.44	19.6	37.26
Significant factors	*A*, *B*, *C*	*A*, *B*, *C*	*A*, *B*, *C*
Observed value of the optimal formula (F4)	80.1	181.5	−40.1
Predicted value of the optimal formula (F4)	78	191.1	−40.4
Absolute deviation%	2.62	5.28	0.75

### 3.2. Model investigation of EE%

**Fig. 2:**
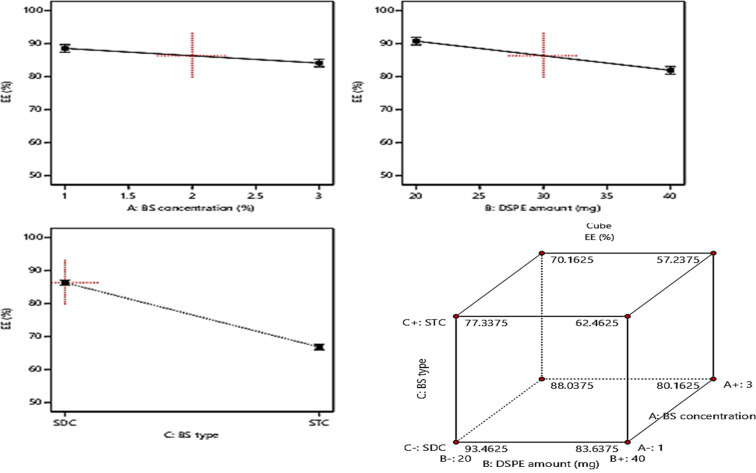
Effect of formulation variables (BS concentration, DSPE amount, and BS type) on EE%.

The determination of EE% of the prepared vesicles can be considered as one of the crucial parameters that highly affect the success of the oral delivery system in delivering a considerable amount of drug to the systemic circulation.^47^ The EE% ranged from (57.3 ± 1.8 and 93.4 ± 2.6%), as displayed in Table 1. The ANOVA results revealed the significant negative impact of the investigated three factors: BS concentration (%w/v) (*A*), DSPE amount (mg) (*B*), and BS type (*C*) on the values of EE%, whereas, the influence of all factors on EE% is graphically displayed in Fig. 2. The resulting equation in terms of coded factors was as follows:EE% = +76.56 − 2.66 × *A* − 5.69 × *B* − 9.76 × *C*

Considering factor (*A*), the increase in the concentration of BS from 1% to 3% resulted in a significant (*p* = 0.0201) decline in EE% and this could be justified by the increased probability of mixed micelles formation with higher BS concentrations which in turn will increase the solubility of the drug in spite of being entrapped within the vesicles formation of mixed micelles.^48^ Moreover, the vesicles fluidity may be increased with higher amounts of BS which results in a subsequent increase in drug leakage rate, thus the EE% will be decreased.^47^ Also increasing the amount of DSPE factor (*B*) resulted in a significant decrease in EE% (*p* = 0.0013) owing to the subsequent interference in the compactness of the vesicular structure and formation of voids in the vesicles on using higher amounts of DSPE.^49^ Besides the increase in the drug solubility that may occur with higher amounts of DSPE is attributed to its high solubilizing power.^50^ Furthermore, ANOVA results revealed that the formulae prepared using SDC possess higher EE% (*p* = 0.0002) than those prepared using STC. This may be due to the larger lipophilic spaces permitted for accommodation of larger amounts of the drug on using BS of higher lipophilicity *i.e.* HLB of SDC (17.6) compared to that of STC (22.1).^15,17^

### 3.3. Model investigation of PS

**Fig. 3:**
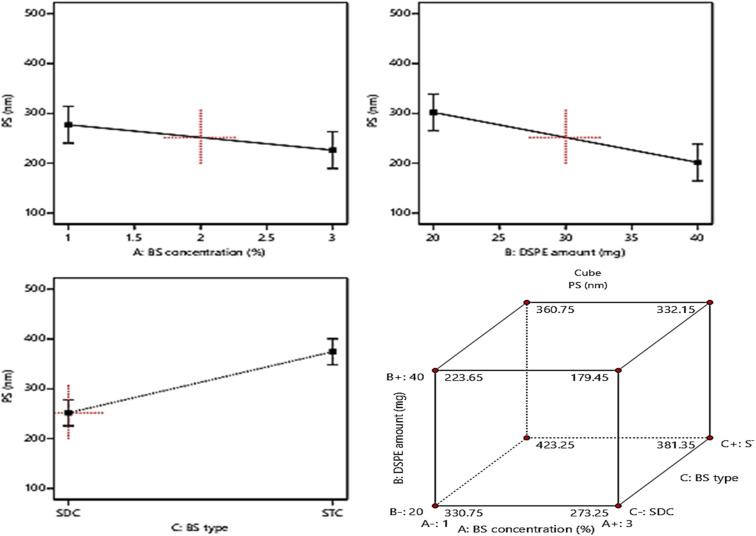
Effect of formulation variables (BS concentration, DSPE amount, and BS type) on PS.

Basically, the ability of the drug molecules to permeate the intestinal membranes is highly correlated to their PS, moreover decreasing the PS, especially to the nano range was found to have a significant positive impact on drug therapeutic activity and duration in systemic circulation and this will lead to the reduction in the required dose and frequent of administration of the drugs.^15,25^ The PS of the fabricated formulae ranged from (181.5 ± 7.2 to 421.2 ± 22.4 nm), Table 1. Based on ANOVA results, it was found that: BS concentration (%w/v) (*A*) and DSPE amount (mg) (*B*), demonstrated a significant positive influence (*p* < 0.05) on PS. On another hand, factor *C* (BS type) exhibited a significant negative influence (*p* < 0.05) on PS. The impact of the three independent variables on PS is graphically illustrated in Fig. 3. The resulting equation in terms of coded factors was as follows:PS = +313.08 − 21.53 × *A* − 39.08 × *B* + 61.3 × *C*With regard to factor *A*: BS concentration (%w/v), it was found that increasing the concentration of BS from 1% to 3% resulted in significant suppression in PS (*p* = 0.0258). This may be attributed to the increased possibility of mixed micelles formation which possess lower PS,^17^ in addition to the subsequent reduction in interfacial tension of the system as a result of increasing the BS concentration leading to a further reduction in PS:^51^ Furthermore, increasing the amount of DSPE (factor *B*) predisposed to a significant reduction in PS (*P* = 0.0033) owing to the subsequent reduction in interfacial tension of the system on using greater amounts of DSPE which will promote the vesicles curvature leading to lower PS.^47^ Also increasing the amount of DSPE with its PEG chains allows the proper wrapping of the bilayer creating steric hindrance at the surface of vesicles and prohibiting their aggregation, hence reducing PS.^21,52^ On the contrary, changing the BS type (factor *C*) resulted in a significant elevation in PS (*p* = 0.0006). This could be explained based on the difference in the molecular weight of STC (537.68 g mol^−1^) compared to SDC (414.6 g mol^−1^) and the bulkiness of the utilized BS.^17^ Additionally, this high molecular weight and greater bulkiness will elevate the viscosity of the dispersion and promote the tendency of the vesicles to agglomerate hence producing larger PS.^49^

### 3.4. Model investigation of PDI

Table 1 revealed that the PDI values of the fabricated vesicles were in the range of ranged from (0.07 ± 0.02 to 0.36 ± 0.08) denoting the homogeneity of the prepared systems. It is well known that the degree of the homogeneity of the dispersion can be concluded from PDI values as the values of PDI getting closer to 0 indicating the homogeneity of the dispersion, on another hand, extensive heterogeneity could be deduced with PDI values approaching 1.^28^ Unfortunately, ANOVA analysis revealed that the three independent variables exhibited a non-significant influence on PDI (*p* > 0.05); so, it wasn't involved in the optimization criteria.

### 3.5. Model investigation of ZP

**Fig. 4:**
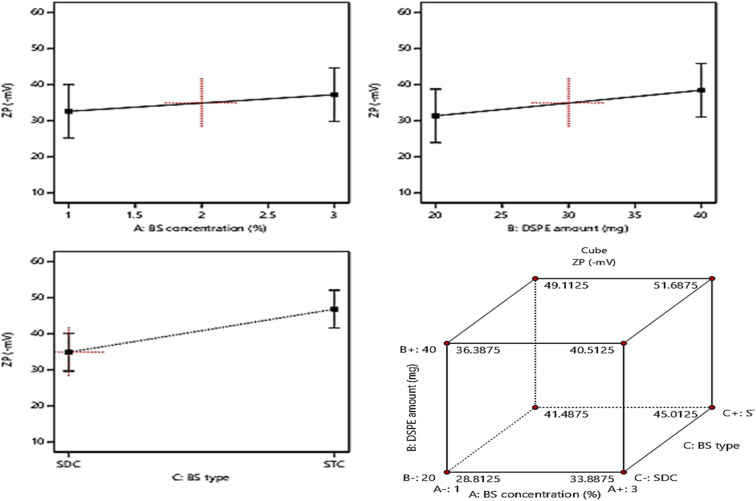
Effect of formulation variables (BS concentration, DSPE amount, and BS type) on ZP.

The allocation of sufficient electro-charges especially on attaining net ZP value in the range of ± 30 mV on the surface of the vesicles assures the stability of the colloidal particles, as these values guarantee the accomplishment of appropriate repulsion forces between the vesicles for prohibiting their tendency for fusion and.^15,52^ The zeta potential values of the fabricated formulae ranged between (−28.4 ± 2.6 to −52.1 ± 4.3), indicating that all the formulae possessed sufficient surface electrostatic charges which assure their stability. The ANOVA results revealed the significant positive impact of the investigated three factors: BS concentration (%w/v) (*A*), DSPE amount (mg) (*B*), and BS type (*C*) on the values of ZP, whereas, the influence of all factors on ZP is graphically displayed in Fig. 4. The resulting equation in terms of coded factors was as follows:ZP = + 40.86 + 1.91 × *A* + 3.56 × *B* + 5.96 × *C*

Considering factor *A*, increasing the amount of bile salt from 1% to 3% significantly increases the ZP as absolute values (*P* = 0.0034), this may be due to the anionic nature of BS which on using greater amounts from them leads to accumulation of higher electronegative charges of the surface of the vesicles, hence increasing the ZP values.^53^ Moreover, ANOVA analysis revealed that the increase in the amount of DSPE predisposed to a significant increase in ZP values (*p* = 0.0003). This could be explained based on the subsequent thickening in the electronegative PEG coat surrounding the bilayers on increasing the amount of anionic DSPE–mPEG which in turn will densify the electronegative charges on the surface of the vesicles and increasing ZP values.^21,54^ It was also noticed that changing the type of BS (factor *C*) from SDC to STC resulted in a significant increase in ZP values (*p* < 0.0001), the difference in number of anionic OH groups between STC (3 OH groups), while SDC (2 OH groups) is behind this prominent increase in absolute ZP values of STC formulae than those of SDC formulae. Moreover, the conjugation of the highly negatively charged taurine group with the bile salt leads to a consequent massive increase in the negative charge of the vesicles containing STC.^49^

### 3.6. Investigation of the validity of the optimization process and choice of the optimal formula

F4 which consists of (3% BS, 40 mg DSPE, and SDC as a bile salt) was selected as the optimum formula with a desirability value of 0.674 using Design Expert® software. Moreover, the optimized formula (F4) showed EE% (80.1 ± 1.9%), PS (181.5 ± 7.2 nm), and ZP (−40.1 ± 4.3 mV) (as absolute value). Noteworthy, % of deviation between the predicted and observed *R*^2^ for all responses (EE%, PS, and ZP) didn't exceed 10% in Table 2, which affirms the suitability of the optimization process and the validity of the whole design.

### 3.7. *In vitro* investigations of the optimum formula

#### 3.7.1. Differential scanning calorimetry (DSC)

**Fig. 5:**
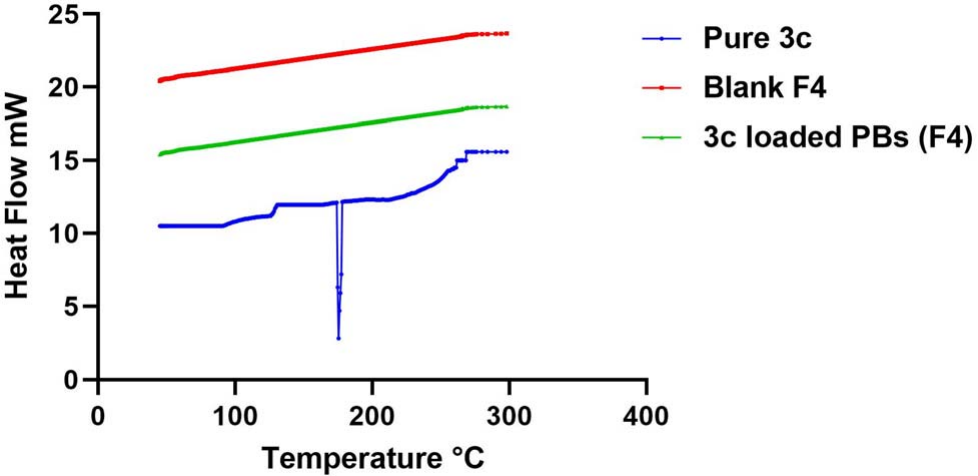
DSC thermograms of pure 3c, blank F4, and 3c-loaded BPs (F4).

The alteration in the degree of crystallinity of the drug “3c” after being loaded in the vesicles had been detected using DSC. Fig. 5 revealed the thermograms of pure “3c” powder compared to that of plain optimized formula and drug-loaded optimized formula (F4). A sharp well well-characterized endothermic peak at 175.4 °C corresponds to the melting point of the drug. On the contrary, this peak vanished in the thermogram of the “3c” loaded optimized formula (F4). Moreover, no characteristic peaks of the formula components could be observed. Thus, it can be concluded that both the drug and the other components were no longer available in the crystalline state and completely transformed to amorphous one which denotes the efficient encapsulation of the drug within the prepared vesicles.^55^

#### 3.7.2. TEM microscopy

**Fig. 6:**
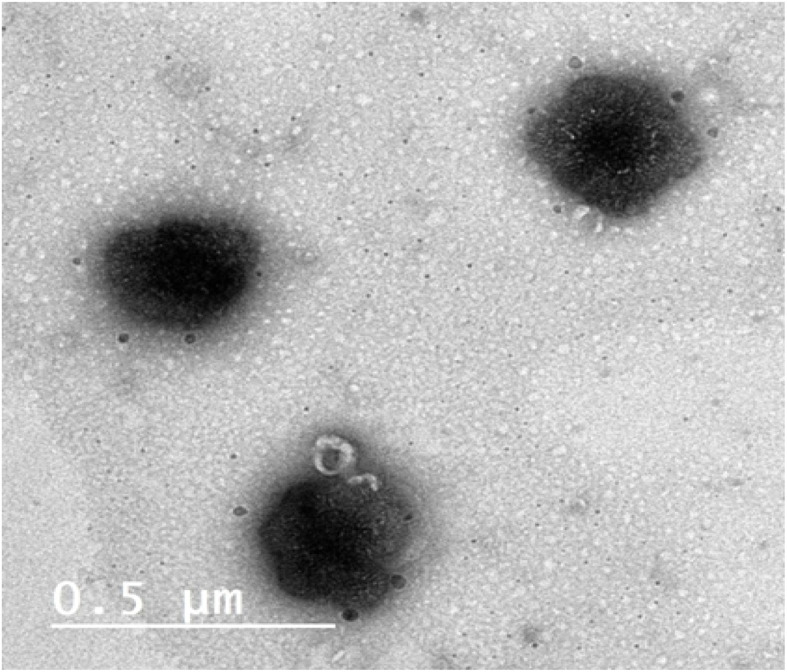
TEM image of optimized formula F4.

The shape of the formulated vesicles of the optimum formula (F4) was detected using TEM. F4 is deemed to be spherical without any abnormalities, Fig. 6. Additionally, the absence of any drug crystals could be observed which indicates the complete inclusion of the drug within the vesicles which empathizes the results of DSC.^56^

#### 3.7.3. *In vitro* drug release

**Fig. 7:**
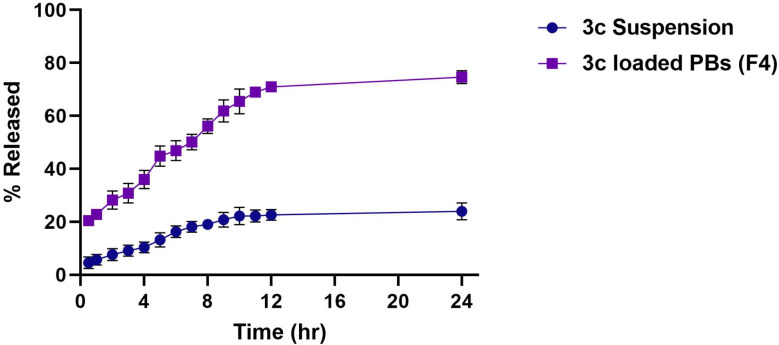
% of drug released from optimized 3c-loaded BPs (F4) *versus*3c suspension.

Fig. 7 revealed the release pattern of “3c” along different time intervals from the optimum formula (F4) compared to the drug dispersion. A biphasic release pattern from F4 could be observed, the first phase distinguished by the burst release of the drug as around 28.6 ± 3.8 of the drug was released from F4 post the first couple of hours compared to 7.8 ± 1.9% released from the drug dispersion owing to the fast release of the free unentrapped drug in the prepared vesicles. On another hand, the second phase was characterized by a sustained and more restricted release of the drug from vesicles reaching 73.3% ± 2.1 for F4 after 24 h compared to 26.8% ± 2.8 from the drug dispersion. This more controlled release was due to the crossing of the drug through the bilayered vesicles and the PEGylated coat that surrounds it. Thus the promoted drug release from F4 could be justified owing to the min PS of the vesicles which increases the surface area subjected to the dissolution medium besides the hydrophilic nature of the PEG coat that surrounds the vesicles which aids in the solubility of the drug and facilitates its contact to the dissolution medium.^15,21,54^

#### 3.7.4. Short-term physical stability study

The optimized formula (F4) was involved in a short-term stability study to assess the impact of storage at 4 °C and 25 °C on its physical characteristics. From Table 3, it can be depicted that no significant change was detected (*p* > 0.05) regarding the assessed parameters (EE%, PS, and ZP) of the optimized formula after being stored compared to those of the freshly prepared formula. The attained steric stability was due to the assembled PEG units as a coat around the vesicles which inhibit the possibility of aggregation and fusion of the vesicles.^15,22,57^

**Table 3:** Effect of storage on the physical characteristics of the optimum 3c-loaded PBs (F4)^a^

Parameter	Fresh F4	Effect of storage at 4 °C on F4	Effect of storage at 25 °C on F4
EE%	80.1% ± 2.8	79.2% ± 1.9	76.2 ± 3.1
PS (nm)	181.5 ± 7.2	186.1 ± 5.1	263.3 ± 11.8
ZP (mV)	−40.1 ± 3.4	−38.7 ± 1.7	−36.7 ± 2.1

a All values were exploited as mean ± SD (*n* = 3).

### 3.8. Comparative *ex vivo* study

**Fig. 8:**
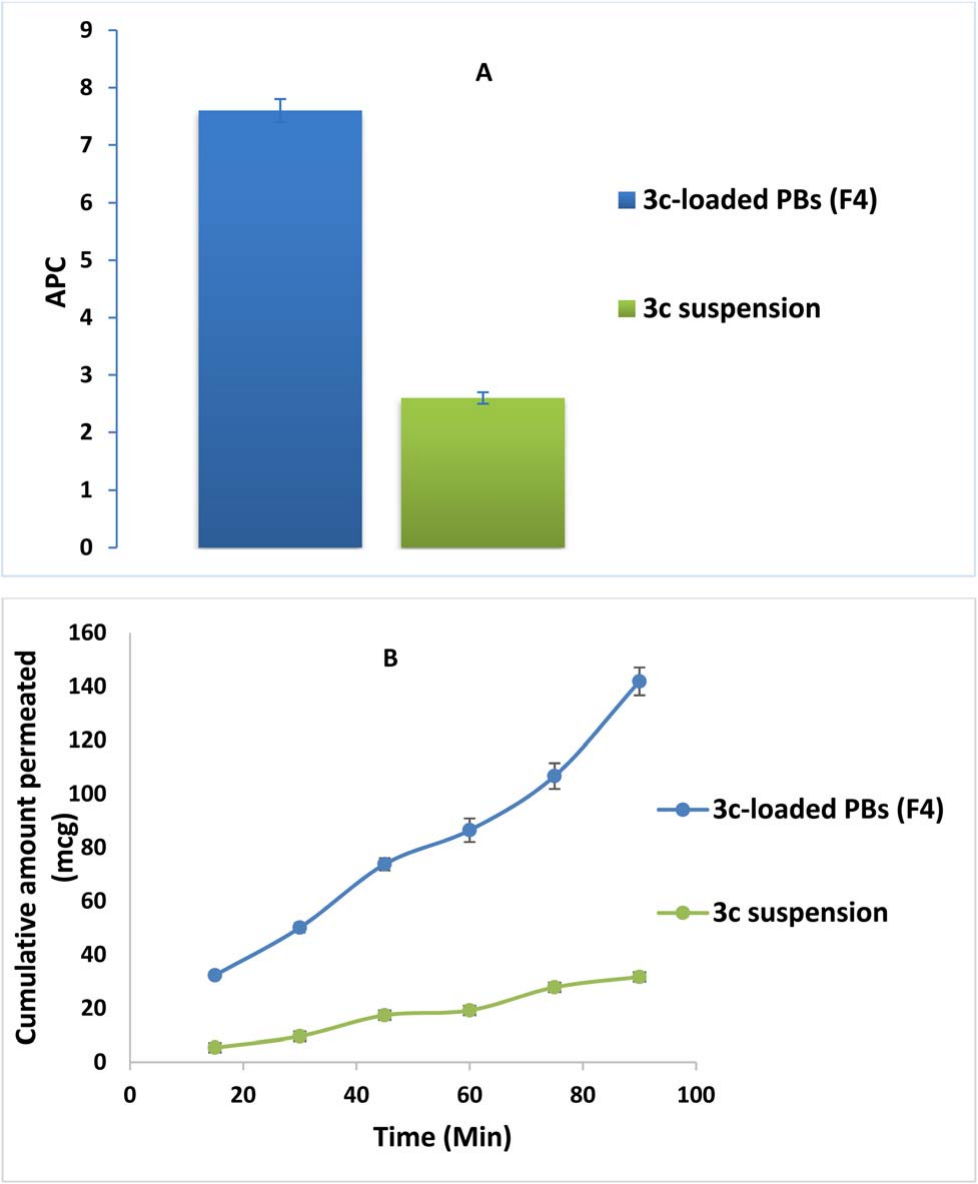
3c*ex vivo* gut permeation study exploiting; (A) *P*_app_ ± S. D. of 3c-loaded PBs (F4) *versus*3c suspension and (B) cumulative amount of 3c permeated (mcg) ± SD of 3c-loaded PBs (F4) *versus*3c suspension time profile.

The effect of formulation on the intestinal permeability of the drug was investigated *via* a comparative *ex vivo* study using a non-everted gut sac. Fig. 8A revealed the superiority of the optimized formula (F4) in the cumulative amount of 3c permeated relative to that of drug suspension at all different time intervals. Moreover, a significant enhancement in the apparent permeability coefficient (APC) of the pure 3c suspension (2.5 × 10^−4^ cm min^−1^) compared to that of the (F4) (7.6 × 10^−4^ cm min^−1^) by around 3 folds (*p* < 0.05) (Fig. 8B). These results may be owing to the minute PS of the vesicles and the solubilizing power of BS and nonionic surfactants used in the compounding of the formula which aids in dissolving the drug and causing disruption of the lipid bilayer of the intestinal membrane, consequently promoting the transcellular and paracellular transport across the intestinal membrane.^58^

### 3.9. *In vitro* studies

#### 3.9.1. SARS-CoV-2 inhibitory assay

Both cytotoxicity and anti-SARS-CoV-2 activity of all examined formulae (PB3a–PB3g) were assessed in Vero E6 cells *via* MTT assay. The half maximal cytotoxic concentrations (CC_50_) were 19.02 μg mL^−1^ (PB3a), 15.8 μg mL^−1^ (PB3b), 8.56 μg mL^−1^ (PB3c), 15.68 μg mL^−1^ (PB3d), 26.63 μg mL^−1^ (PB3e), 18.27 μg mL^−1^ (PB3f), and 12.16 μg mL^−1^ (PB3g). In addition, the half maximal inhibitory concentrations (IC_50_) were 5.9 μg mL^−1^ (PB3a), 5.02 μg mL^−1^ (PB3b), 0.71 μg mL^−1^ (PB3c), 1.30 μg mL^−1^ (PB3d), 3.18 μg mL^−1^ (PB3e), 2.25 μg mL^−1^ (PB3f), and 1.25 μg mL^−1^ (PB3g).

**Fig. 9:**
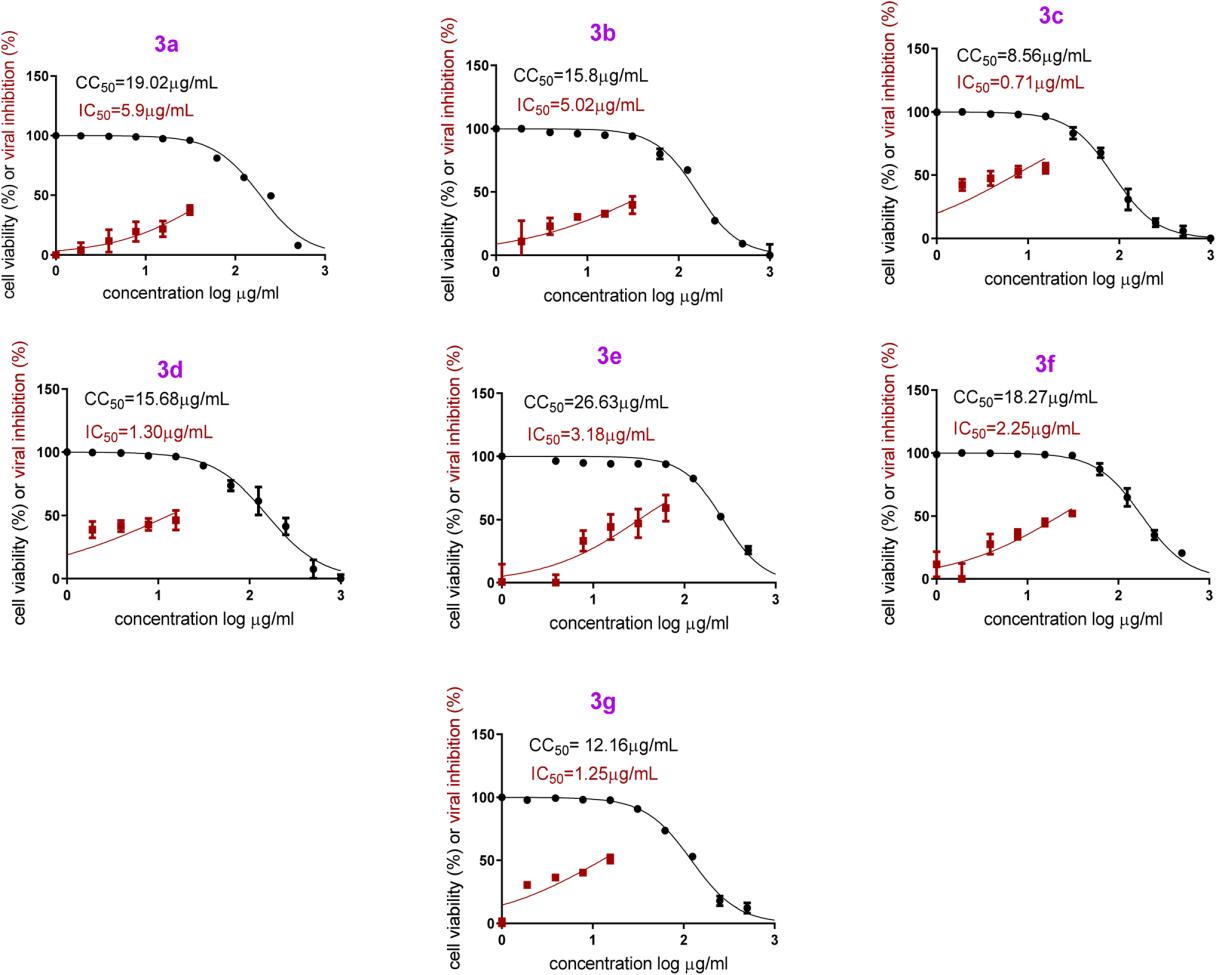
*In vitro* anti-SARS-CoV-2 activity and cytotoxicity of PB3a–PB3g in Vero E6 cells *via* MTT assay [*h*CoV-19/Egypt/NRC-03/2020 (Accession Number on GSAID: EPI_ISL_430820)].

On the other hand, both PB3c and PB3g displayed the highest IC_50_ values ensuring their superior antiviral potential. Besides, PB3e revealed the highest CC_50_ value ensuring its safety on normal cells, Fig. 9. The highest selectivity index (SI = CC_50_/IC_50_) was shown by formulas PB3c and PB3d at values of 12.05 and 12.06, respectively.

The improved antiviral effectiveness of the compounds following their formulation can be attributed to the ability of the customized vesicles to attach to viral cells through either endocytosis or fusion mechanisms. This attachment facilitated the accumulation of the compounds around the viral membrane, creating concentration and thermodynamic gradients that favored the accumulation and boosted the penetration of those compounds through the virus's cellular membranes. Additionally, the lipids and surfactants integrated into the PB formulation played a crucial role in enhancing the antiviral activity of the compounds by promoting their diffusion through the viral cells.^30^

#### 3.9.2. Mode of action against SARS-CoV-2

**Fig. 10:**
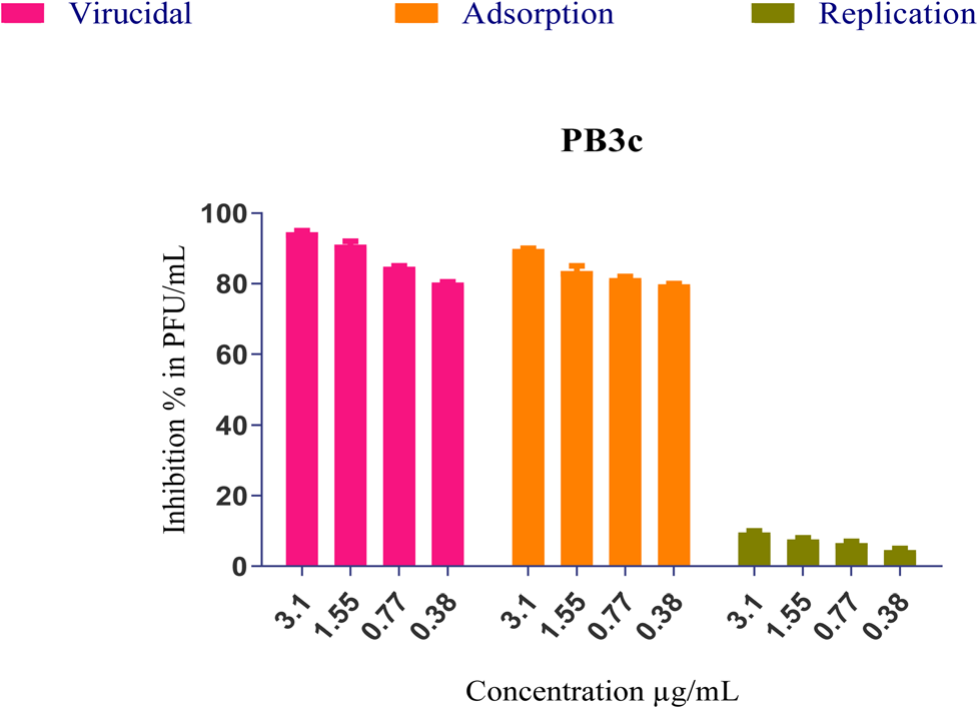
The mechanism of action of the most potent formulation (PB3c) against SARS-CoV-2.

The most effective formulated compound against SARS-CoV-2 (PB3c) was chosen for in-depth exploration of its potential mode of action. To investigate this, a range of four safe concentrations was used, and three potential anti-viral mechanisms were studied: direct virucidal activity, prevention of viral adsorption to host cell receptors, and inhibition of intracellular viral replication. The findings revealed that PB3c demonstrated a noteworthy combination of actions, including more than 80% virucidal activity and over 80% inhibition of viral adsorption as the primary mechanisms targeting SARS-CoV-2 spike protein. However, much little effect on viral replication was shown at ∼(5–10%), Fig. 10.

### 3.10. *In silico* studies

#### 3.10.1. Docking study

**Fig. 11:**
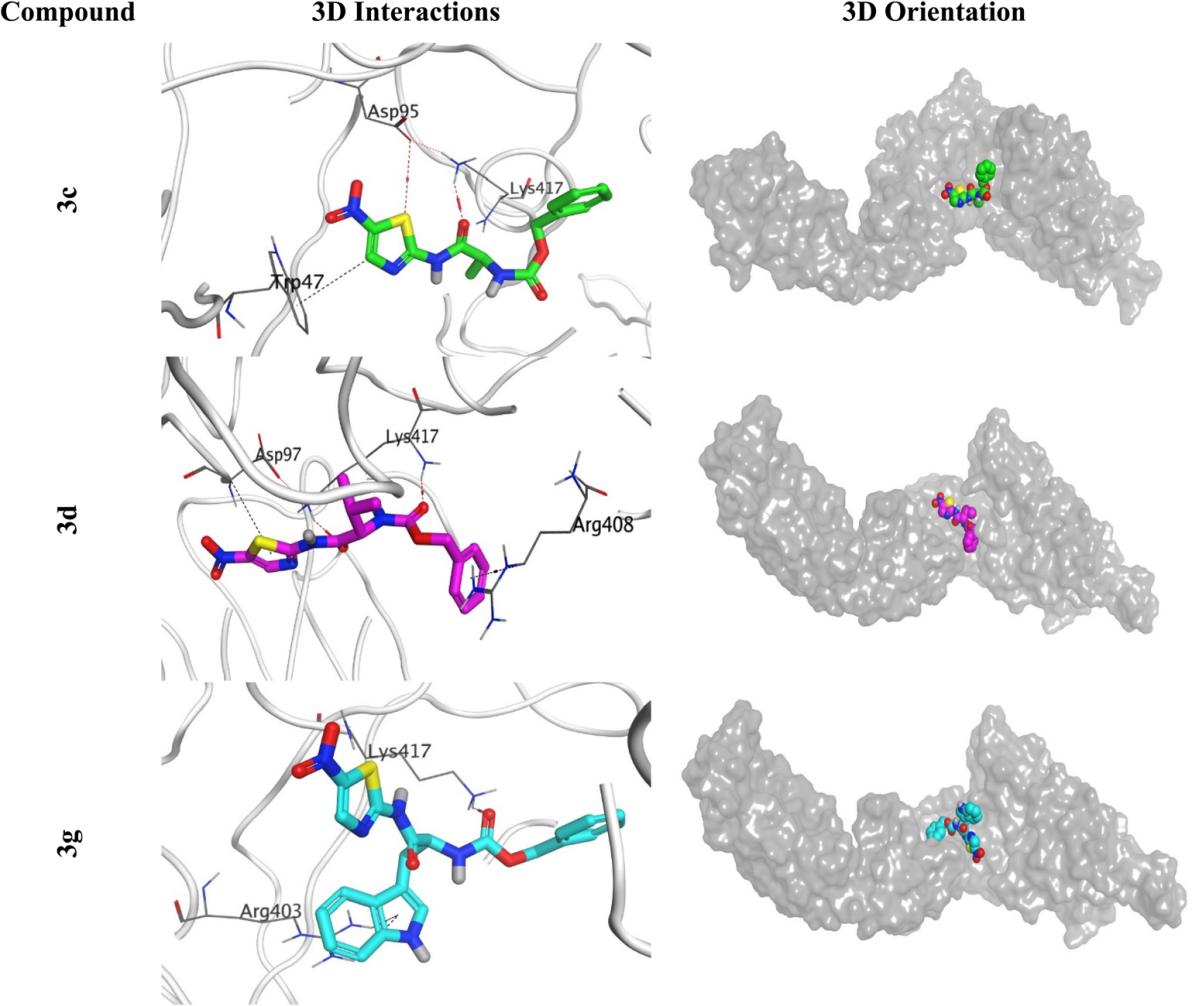
3D Binding interactions and orientations of compounds (3c, 3d, and 3g) within the SARS-CoV-2 spike protein receptor (PDB ID: 6XE1).

The docking study of the seven examined candidates (3a, 3b, 3c, 3d, 3e, 3f, and 3g) towards the SARS-CoV-2 spike protein receptor binding domain (PDB ID: 6XE1) clarified that 3c, 3d, and 3g members were the most promising ones. Their binding scores were recorded to be −6.47, −6.55, and −7.15 kcal mol^−1^ with Root Mean Square Deviation (RMSD) values of 1.79, 1.97, and 1.39 Å, respectively. Compound 3c showed the formation of two hydrogen bonds with ASP95 and LYS417 and one hydrogen–pi bond with TRP47. Besides, compound 3d formed one hydrogen bond with LYS417 and two pi–hydrogen bonds with ASP97 and ARG408. However, compound 3g interactions were recorded as one hydrogen bond with LYS417 and two pi–cation bonds with ARG403 (Fig. 11).

#### 3.10.2. Molecular dynamics studies

The behaviour of the seven docked complexes (3a, 3b, 3c, 3d, 3e, 3f, and 3g) towards the SARS-CoV-2 spike protein receptor binding domain was described through MD simulations for 150 ns.

##### 3.10.2.1. RMSD analysis

**Fig. 12:**
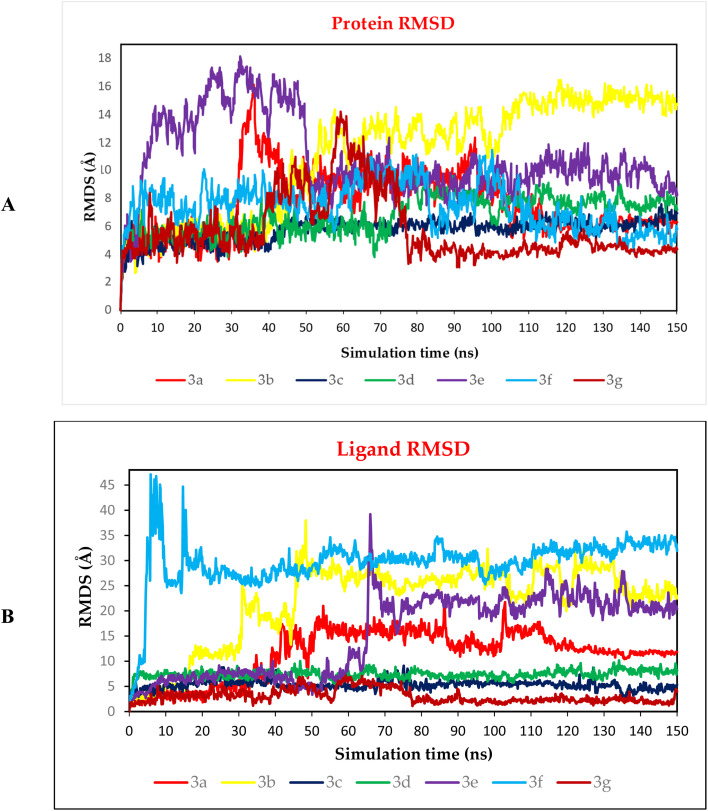
(A) Protein RMSD and (B) ligand RMSD for complexes (3a, 3b, 3c, 3d, 3e, 3f, and 3g) of SARS-CoV-2 spike protein receptor binding domain as a function of simulation time (150 ns).

To be able to judge the system's stability; the RMSD was performed. The RMSD could describe the deviation degree of the complex protein with respect to its initial position in a quantitative way. The seven examined complexes showed moderate stability with the best behaviour for 3c, 3d, and 3g complexes (Fig. 12A), these three complexes showed an RMSD that is more stable compared to other complexes, in which these complexes showed a high fluctuation. On the other hand, the ligand RMSD (L-RMSD) was studied to evaluate the exact behaviour of each ligand within the SARS-CoV-2 spike protein receptor binding domain (Fig. 12B). Compounds 3c, 3d, and 3g showed the most promising behaviours with RMSD values of 9, 10, and 6.5 Å, as a maximum fluctuation level, respectively, this indicated great stability in binding to the receptor binding domain, which may suggest that these compounds mechanism of action involve the inhibition of the SARS-Cov-2 spike protein. Notably, compound 3c fluctuated around 6 Å most of the simulation time and reached 9 Å only for small periods indicating a stable behaviour. Moreover, compound 3d was nearly stable in its fluctuations all over the simulation time around 8 Å indicating a highly stable behaviour as well. However, the fluctuations of compound 3g were around 3 Å most of the simulation time except from (40–75) Å where it reached 6.5 Å indicating a highly promising and stable behaviour inside the target receptor pocket.

##### 3.10.2.2. Binding interactions histogram and heat map analysis

**Fig. 13:**
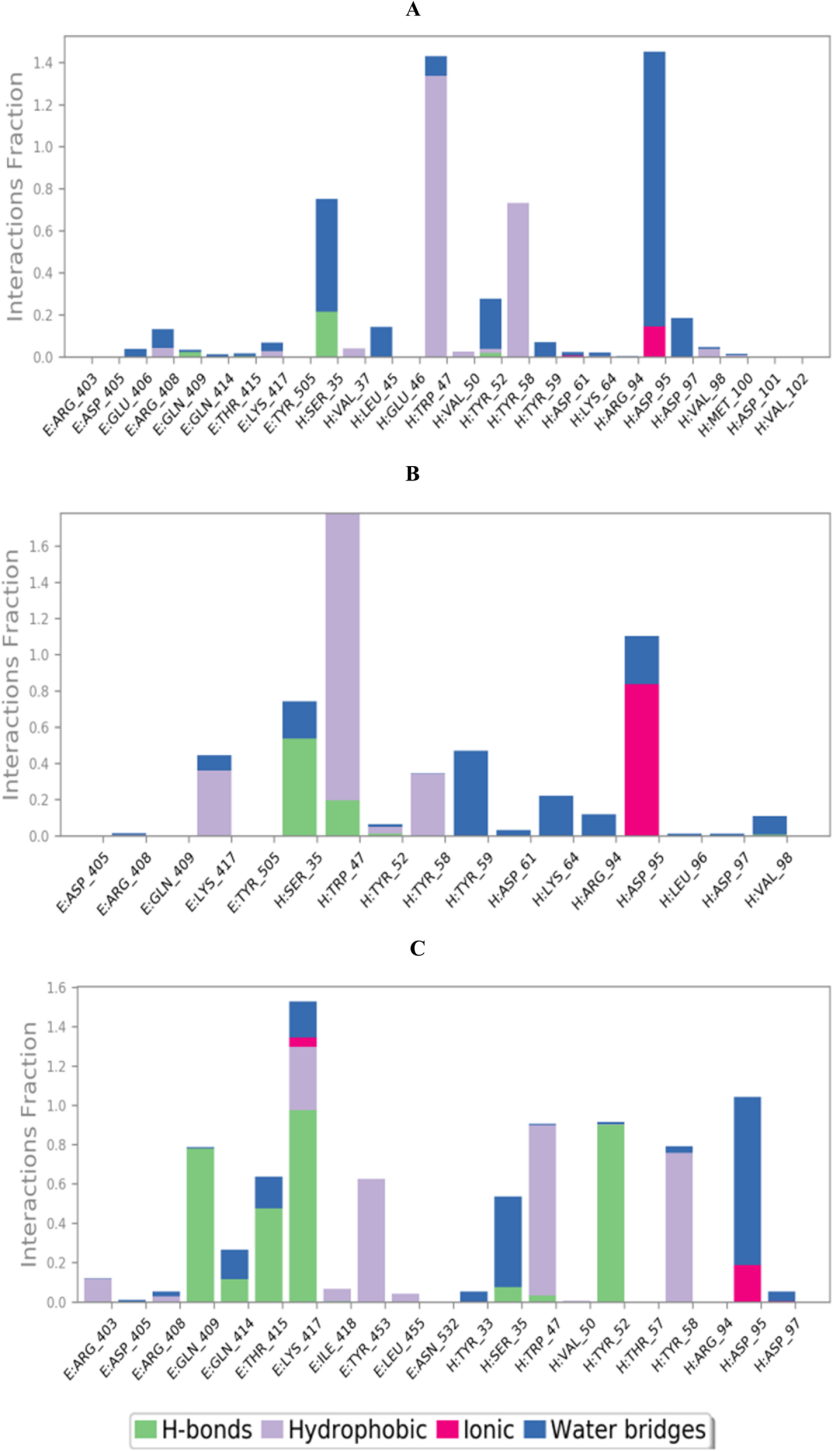
Histogram describing the binding interactions between the SARS-CoV protein and its ligand during the simulation time of 100 ns for (A) 3c, (B) 3d, and (C) 3g.

**Fig. 14:**
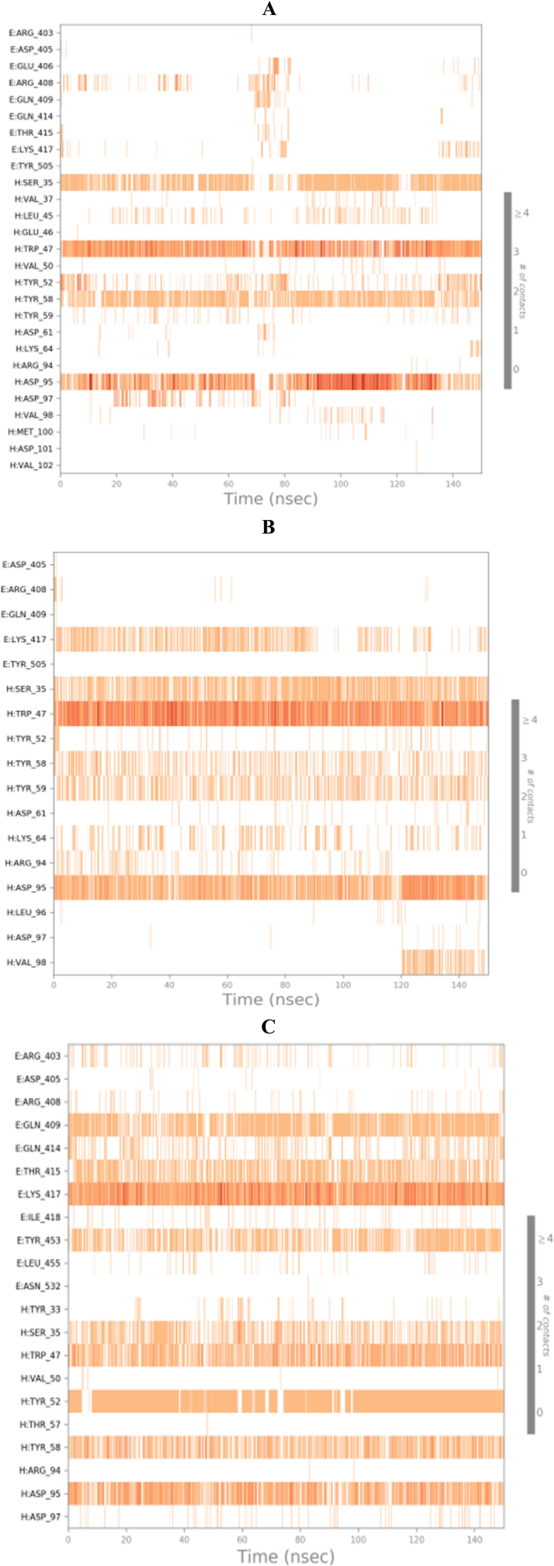
Heat map showing the total number of SARS-CoV protein–ligand interactions all over the simulation time of 100 ns for (A) 3c, (B) 3d, and (C) 3g.

To completely describe the SARS-CoV-2 spike protein-ligand interactions; both histograms and heat maps were discussed in detail (Fig. 13 and 14).

The histogram of the SARS-CoV-2 spike protein in complex with compound 3c showed that both ASP95 and TRP47 were the most contributing amino acids in the interactions with about 140%. The types of interactions were (water bridges and ionic) and (hydrophobic and water bridges) for ASP95 and TRP47, respectively (Fig. 13A). Moreover, for compound 3d; the same two amino acids (ASP95 and TRP47) were the Frontier contributing ones with 100 and 180%, respectively. Notably, the (ionic and water bridges) and (hydrophobic and hydrogen bonds) were the types of interactions for ASP95 and TRP47, respectively (Fig. 13B). However, the SARS-CoV-2 spike protein–3g histogram represented that ASP95 and LYS417 were the superior amino acids with 100 and 150% contributions through (water bridges and ionic) and (hydrogen bonds, hydrophobic, water bridges, and ionic) types, respectively (Fig. 13C).

Briefly, it could be concluded that ASP95, TRP47, and LYS417 amino acids are the most crucial residues to be bound to produce the inhibitory potential towards the SARS-CoV-2 spike protein binding domain.

On the other side, the exact time of the binding interactions for the SARS-CoV-2 spike protein residues with 3c, 3d, and 3g candidates were represented by heat map output (Fig. 14).

The heat map of the SARS-CoV-2 spike protein-3c complex described that both ASP95 and TRP47 interactions were along the simulation time and showed the lowest contributions for ASP95 at the middle and the end of the simulation (Fig. 14A). Furthermore, more intense behaviour was observed for ASP95 and TRP47 interactions with compound 3d all over the 150 ns of the simulation (Fig. 14B). Besides, the SARS-CoV-2 spike protein–3g complex heat map showed that ASP95 and LYS417 were continually contributing to the binding interactions from the start until the end of the simulation time (Fig. 14C).

### 3.11. MM-GBSA calculations

The MM-GBSA calculations using the thermal_mmgbsa.py python script of Schrodinger^38,59^ were carried out for all complexes (3a, 3b, 3c, 3d, 3e, 3f, and 3g) and represented in Table 4. Notably, compound 3g achieved the superior Δ*G* binding energy (−63.37 kcal mol^−1^), followed by 3e, 3f, 3d, and 3c with binding energy scores of −43.31, −43.20, −42.62, and −38.36 kcal mol^−1^, respectively.

**Table 4:** MM-GBSA energies for complexes (3a, 3b, 3c, 3d, 3e, 3f, and 3g) of SARS-CoV spike protein^a^

Complex	Δ*G* binding	Coulomb	Covalent	H-bond	Lipo	Bind packing	Solv_GB	VdW	St dev.
3a	−33.64	3.33	1.16	−0.61	−10.85	−3.61	4.77	−27.83	8.51
3b	−33.91	−5.15	1.66	−0.86	−8.00	−2.10	8.60	−28.05	7.63
3c	−38.36	2.19	1.06	−0.28	−12.44	−4.88	8.27	−32.29	4.19
3d	−42.62	1.97	−0.01	−0.47	−14.46	−5.61	9.748	−33.77	3.53
3e	−43.31	−5.29	2.62	−0.92	−13.34	−3.21	10.95	−34.12	10.48
3f	−43.20	−5.18	0.76	−0.53	−11.98	−0.40	9.47	−35.34	11.27
3g	−63.37	−17.34	5.36	−2.09	−16.08	−7.23	22.89	−48.87	4.85

a Coulomb: Coulomb energy; covalent: covalent binding energy; H-bond: hydrogen-bonding energy; Lipo: lipophilic energy; Solv_GB: generalized born electrostatic solvation energy; VdW: van der Waals energy; and St dev.: standard deviation.

## 4 Structure–activity relationship (SAR) study of the examined formulae (PB3a–PB3g)

**Fig. 15:**
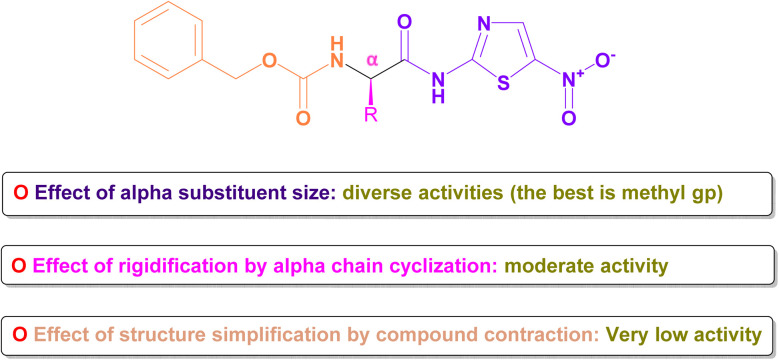
Summary of SAR studies for the synthesized *N*-(5-nitrothiazol-2-yl)-carboxamido derivatives as potential inhibitors of SARS-CoV-2.

The core structure, *N*-(5-nitrothiazol-2-yl) carboxamide, remained constant across all compounds investigated. Our primary emphasis was on investigating how modifying the size, type, and flexibility of the α-substituent attached to the carboxamide, along with compound contraction, affected the activity against SARS-CoV-2. These investigations were based mainly on the IC_50_ values of the assessed formulae (PB3a–PB3g) against SARS-CoV-2. The conducted SAR studies for the synthesized *N*-(5-nitrothiazol-2-yl)-carboxamido derivatives as potential inhibitors of SARS-CoV-2 were summarized in Fig. 15.

### 4.1. Effect of α-substituent size

Generally, it was concluded that there is no direct correlation between the α-substituent size and the inhibitory potential attained against SARS-CoV-2. For example, it was revealed that the best inhibitory potential was attained by substituting α-carbon attached to the carboxamide with the small-size methyl group (3c). However, un-substitution of the α-carbon (3b) or substitution with more bulky groups such as isopropyl (3d), benzyl (3e), 1*H*-indol-3-yl *via* methylene bridge (3g) displayed much lower inhibitory potential against SARS-CoV-2.

### 4.2. Effect of rigidification by α-chain cyclization

One frequently employed approach in drug design is the incorporation of chain cyclization to induce rigidity. This strategy is widely used as it can potentially improve binding affinity and/or stabilize target binding interactions.^60^ Interestingly, it was disclosed that forming the conformationally-restricted pyrrolidine moiety by cyclizing the NH of benzyl carbamate with the α-carbon *via* a propyl spacer (3f) afforded better inhibitory potential against SARS-CoV-2 than unsubstituted α-carbon derivative (3b) and benzyl substituted α-carbon derivative (3e). However, the cyclization approach displayed lower inhibitory potential against SARS-CoV-2 than substituted α-carbon derivatives with methyl (3c), isopropyl (3d), or 1*H*-indol-3-yl *via* methylene bridge (3g).

### 4.3. Effect of structure simplification by compound contraction

Structure simplification is one of the important tools of lead optimization in drug design which aims mainly to reduce side effects and facilitate chemical synthesis saving money and time.^61^ It was shown that structure simplification (compound contraction) *via* removing the methylcarbamate moiety and directly acylating the aminothiazole scaffold with a tolyl ring (3a) made, unfortunately, a remarkable decrease in the inhibitory potential against SARS-CoV-2 among all afforded compounds. So, we can urge that the decreased activity may be attributed to the importance of the removed moiety for the binding interaction with the SARS-CoV-2 spike.

## 5 Conclusion

In the present study, PBs were developed in order to boost the anti-SARS-CoV-2 activity of the examined chemical compounds. The formulae were constructed based on 2^3^ full factorial design considering all the combinations of the two levels of each independent variable. Then F4 was selected as the optimum formula, whereas, its composition (3% (BS), 40 mg (DSPE), and SDC as a bile salt) was utilized in the formulation of all compounds loaded PBs. The newly formulated compounds exhibited promising SARS-CoV-2 inhibitory potential, in particular, for formulated compounds 3c and 3g. In addition, the conducted mechanistic study could assure the promising inhibitory potential of PB3c with more than 80% virucidal activity and over 80% inhibition of viral adsorption through SARS-CoV-2 spike protein and ACE2 receptor inhibition. Moreover, the conducted molecular docking and dynamics declared the eligible binding interactions of the investigated compounds with reasonable stability behavior during the simulation time at SARS-CoV-2 spike protein. Besides, the established SAR studies declared that there is no direct correlation between the α-substituent size and the inhibitory potential attained against SARS-CoV-2. However, structure rigidification may afford moderate activity, whereas, structure simplification could lead to activity drop. Accordingly, based on the aforementioned data pegylated bilosomes could be used as prosperous nano-carriers for drugs with boosted anti-SARS-CoV-2 activity.

## Data availability

All data generated or analyzed during this study are included in this published article and its ESI.†

## Author contributions

Conceptualization and supervision: Mohamed Y. Zakaria and Ahmed A. Al-Karmalawy; data curation: Mohamed Y. Zakaria, Ayman Abo Elmaaty, Radwan Alnajjar, Sana Ben Moussa, Abdullah Yahya Abdullah Alzahrani, Ahmed A. Al-Karmalawy; visualization: Mohamed Y. Zakaria, Ayman Abo Elmaaty, Rabeh El-Shesheny, Radwan Alnajjar, Omnia Kutkat, Ahmed A. Al-Karmalawy; methodology: Mohamed Y. Zakaria, Ayman Abo Elmaaty, Rabeh El-Shesheny, Radwan Alnajjar, Omnia Kutkat, Sally A. El-Zahaby and Ahmed A. Al-Karmalawy; writing – review & editing: Mohamed Y. Zakaria, Sally A. El-Zahaby, Ayman Abo Elmaaty, Rabeh El-Shesheny, Sana Ben Moussa, Abdullah Yahya Abdullah Alzahrani, and Ahmed A. Al-Karmalawy. Finally, all authors revised and approved the final submitted manuscript.

## Conflicts of interest

The authors declared no conflict of interest.

## Supplementary Material

RA-014-D4RA07316A-s001
