# Supplementary material for: Biological and computational assessments of thiazole derivative-reinforced bile salt enriched nano carriers: a new gate in targeting SARS-CoV-2 spike protein
Source: RSC Adv. 2024 Dec 9;14(52):38778–95. doi: 10.1039/d4ra07316a (PMC11627215; doi:10.1039/d4ra07316a)
Supplement: RA-014-D4RA07316A-s001 [file RA-014-D4RA07316A-s001.pdf]

Supplementary Information (SI) for RSC Advances.
This journal is © The Royal Society of Chemistry 2024

Supplementary Materials

Biological and Computational Assessments of Thiazole Derivatives-Reinforced Bile Salt Enriched Nano Carriers: A New Gate in Targeting SARS-CoV-2 Spike Protein

Mohamed Y. Zakaria[1,2,*], Ayman Abo Elmaaty[3,4], Rabeh El-Shesheny[5], Radwan Alnajjar[6,7], Omnia Kutkat[5], Sana Ben Moussa[8], Abdullah Yahya Abdullah Alzahrani[8], Sally A. El-Zahaby[9], Ahmed A. Al-Karmalawy[10,11,*]

[1] Department of Pharmaceutics and Industrial Pharmacy, Faculty of Pharmacy, King Salman International University, Ras Sudr 46612, South Sinai, Egypt.
[2] Department of Pharmaceutics and Industrial Pharmacy, Faculty of Pharmacy, Port Said University, Port Said 42526, Egypt.
[3] Medicinal Chemistry Department, Faculty of Pharmacy, Port Said University, Port Said 42526, Egypt.

[4] Medicinal Chemistry Department, Clinical Pharmacy Program, East Port said National University, Port Said 42526, Egypt.

[5] Excellence for Influenza Viruses, Water Pollution Research Department, Environmental Research Institute, National Research Centre, Dokki-Giza 12622, Egypt.
[6] CADD Unit, Faculty of Pharmacy, Libyan International Medical University, Benghazi 16063, Libya.

[7] Department of Chemistry, Faculty of Science, University of Benghazi, Benghazi 16063, Libya.

[8] Department of Chemistry, Faculty of Science and Arts, King Khalid University, Mohail Assir, Saudi Arabia.

[9] Department of Pharmaceutics and Industrial Pharmacy, PharmD Program, Egypt-Japan University of Science and Technology (E-Just), Alexandria, Egypt.

[10] Department of Pharmaceutical Chemistry, College of Pharmacy, The University of Mashreq, Baghdad 10023, Iraq.

[11] Department of Pharmaceutical Chemistry, Faculty of Pharmacy, Horus University-Egypt, New Damietta 34518, Egypt.

*Correspondence:

Ahmed A. Al-Karmalawy; Email: akarmalawy@horus.edu.eg

Mohamed Y. Zakaria; Email: mohamed.yehia@ksiu.edu.eg

Materials and Methods

S1. MTT cytotoxicity assay (CC_{50})

Samples were diluted with Dulbecco's Modified Eagle's Medium (DMEM). Stock solutions of the test compounds were prepared in 10 % DMSO in dd H_2O. The cytotoxic activity of the samples were tested in Vero-E6 cells by using the 3-(4, 5-dimethylthiazol-2-yl)-2, 5-diphenyltetrazolium bromide (MTT) method with minor modification [1]. Briefly, the cells were seeded in 96 well-plates (100 µL/well at a density of 3×105 cells/mL) and incubated for 24 h at 37 °C in 5% CO_2. After 24 h, cells were treated with various concentrations of the tested samples in triplicates. After a further 72 h, the supernatant was discarded and cell monolayers were washed with 1X sterile phosphate buffer saline (PBS) 3 times and MTT solution (20 µL of 5 mg/mL stock solution) was added to each well and incubated at 37 °C for 4 h followed by medium aspiration. In each well, the formed formazan crystals were dissolved with 200 µL of DMSO (0.04 M HCl in absolute isopropanol = 0.073 mL HCl in 50 mL isopropanol). The absorbance of formazan solutions was measured at λmax 540 nm with 620 nm as a reference wavelength using a multi-well plate reader. The plot of % cytotoxicity versus sample concentration was used to calculate the concentration which exhibited 50% cytotoxicity (TC_{50}). The % of cytotoxicity compared to the untreated cells was determined with the following equation:

$$\% \, cytotoxicity = \frac{(absorbance\ of\ cells\ without\ treatment - absorbance\ of\ cells\ with\ treatment) X 100}{absorbance\ of\ cells\ without\ treatment}$$

S2. Inhibitory concentration 50 (IC_{50}) determination

The Vero-E6 cells (2.4×104) were kept overnight at 37°C in 5% CO_2 inside 96-well tissue culture plates. 1x PBS solution was used to wash the cell monolayers for only one time which were then treated with different serial dilutions of the examined compounds together with a fixed dilution from the virus (hCoV-19/Egypt/NRC-03/2020 (Accession Number on GSAID: EPI_ISL_430820)) following TCID50 test and kept at RT for 1 h before starting incubation. Also, the cell monolayers were subjected to DMEM (100 µl) with different concentrations of the test samples and virus and left at 37°C for 72 h in a 5% CO2. Then, 4% paraformaldehyde (100 µl) was used for cell fixation (2 h) followed by the staining step with 0.1% crystal violet in distilled H2O (50 µl) at RT for 15 min. Absolute CH3OH (100 µl) was added to dissolve the crystal violet dye per well to measure the optical density of the produced color using Anthos Zenyth 200rt plate reader at 570 nm.[2] The IC_{50} value for each tested compound which is corresponding to

its minimum concentration required to reduce the virus infectivity by 50% in comparison to the virus control was calculated.

S3. Mode of action of virus inhibition

The possible mode of action of virus inhibition by the F4 was examined at three different stages of the virus propagation cycle and based on three main possible modes of action: (i) Inhibition of budding and viral replication. (ii) The ability of each formula to inhibit of attachment of the virus to infected cells-membrane fusion known as blocking the viral entry (viral adsorption); and (iii) The direct effect of each formula to inactivate the virus viability (virucidal activity). Additionally, the above-mentioned mode of action could account for the recorded antiviral activities either independently, or in combinations. In this regard, the interaction between the formulae and the MERS-CoV virus could be explained through the following three different modes of action:

S3.1. Virucidal

The virucidal assay was carried out [3] in a 6 wells plate where Vero-E6 cells were cultivated (105 cells/mL) for 24 h at 37 ^{o}C. A volume of 200 µL serum-free DMEM containing virus was added to the concentration of the tested formula. After 1 h incubation, the mixture was diluted using serum-free medium 3 times each 10-fold which still allows the existence of viral particles to grow on Vero-E6 cells but leaves nearly no extract and 100 µL of each dilution was added to the Vero-E6 cell monolayer. After 1 h contact time, DMEM over layer was added to cell monolayer. Plates were left to solidify and then incubated at 37 ^{o}C to allow the formation of viral plaques, fixed and stained as above mentioned to calculate percentage reduction in plaques formation in comparison to control wells where cells were infected with the virus that was not pretreated with the tested formulae.

S3.2. Viral adsorption

Vero-E6 cells were cultivated in a 6 wells plate (105 cells/mL) for 24 h at 37 ^{o}C for the viral adsorption assay using the Zhang et al. method [4]. Each formula was applied at different concentrations in a 200 µL medium without supplements and co-incubated with the cells for 2 h at 4 ^{o}C. The unabsorbed formula was removed by washing cells 3 successive times with supplements free-medium then virus diluted was co-incubated with the pretreated cells for 1 h followed by adding 3 mL DMEM supplemented with 2% agarose. Plates were left to solidify and then incubated at 37 ^{o}C to allow the formation of viral plaques, fixed and stained as above mentioned to calculate percentage reduction in plaques formation in comparison to control wells where untreated Vero-E6 cells were directly infected with the virus.

S3.3. Viral replication

The viral replication assay was carried out according to Kuo et al. [5] in a 6-well plate where VERO-E6 cells were cultivated (105 cells/mL) for 24 h at 37 ^{o}C. The virus was diluted to 103 PFU/well and applied directly to the cells and incubated for 1 h at 37 ^{o}C. Unabsorbed viral particles were removed by washing cells 3 successive times with supplements free-medium. The formula was applied at different concentrations, after 1 h contact time, add 3 mL of 2X DMEM medium supplemented with 2% agarose to the cell monolayer. Plates were left to solidify and incubated at 37 ^{o}C till the appearance of viral plaques. Cell monolayers were fixed in 10% formalin solution for 2 h, and stained with crystal violet. Control wells were included where Vero-E6 cells were incubated with the virus and didn't treat with the extract. Finally, plaques were counted and percentage reduction in plaques formation in comparison to control wells was recorded as above mentioned.

S4. Molecular dynamics simulations

The MD simulations were carried out using Desmond simulation package of Schrödinger LLC.[6] The NPT ensemble with the temperature 300 K and a pressure 1 bar was applied in all runs. The simulation length was 150 ns with a relaxation time 1 ps for the ligands. The OPLS3 force field parameters were used in all simulations.[7] The cutoff radius in Coulomb interactions was 9.0 Å. The orthorhombic periodic box boundaries were set 10 Å away from the protein atoms. The water molecules were explicitly described using the transferable intermolecular potential with three points (TIP3P) model.[8, 9] Salt concentration set to 0.15 M NaCl and was built using the System Builder utility of Desmond.[10] The Martyna−Tuckerman−Klein chain coupling scheme with a coupling constant of 2.0 ps was used for the pressure control and the Nosé−Hoover chain coupling scheme for the temperature control.[11, 12] Nonbonded forces were calculated using a RESPA integrator where the short-range forces were updated every step and the long-range forces were updated every three steps. The trajectories were saved at 20 ns intervals for analysis. The behavior and interactions between the ligands and protein were analyzed using the Simulation Interaction Diagram tool implemented in Desmond MD package. The stability of MD simulations was monitored by looking on the RMSD of the ligand and protein atom positions in time.

S5. MD trajectory analysis and prime MM-GBSA calculations

Simulation interactions diagram panel of Maestro software was used to monitoring interactions contribution in the ligand-protein stability. The molecular mechanics generalized born/solvent accessibility (MM – GBSA) was performed to calculate the ligand binding free energies and ligand strain

energies for docked compounds over the last 25 ns with thermal_mmgbsa.py python script provided by Schrodinger which takes a Desmond trajectory file, splits it into individual snapshots, runs the MM-GBSA calculations on each frame, and outputs the average computed binding energy.

References

1. R. Alnajjar, A. Mostafa, A. Kandeil and A. A. Al-Karmalawy, *Heliyon*, 2020, **6**, e05641.
2. N. P. Marques, C. S. Lopes, N. C. T. Marques, L. Cosme-Silva, T. M. Oliveira, C. Duque, V. T. Sakai and J. A. C. Hanemann, *Lasers in medical science*, 2019, **34**, 465-471.
3. A. Schuhmacher, J. Reichling and P. Schnitzler, *Phytomedicine : international journal of phytotherapy and phytopharmacology*, 2003, **10**, 504-510.
4. J. Harcourt, A. Tamin, X. Lu, S. Kamili, S. K. Sakthivel, J. Murray, K. Queen, Y. Tao, C. R. Paden and J. Zhang, *Emerging infectious diseases*, 2020, **26**, 1266.
5. Y. C. Kuo, L. C. Lin, W. J. Tsai, C. J. Chou, S. H. Kung and Y. H. Ho, *Antimicrobial agents and chemotherapy*, 2002, **46**, 2854-2864.
6. S. Release, *Maestro-Desmond Interoperability Tools, Schrödinger, New York, NY*, 2017.
7. E. Harder, W. Damm, J. Maple, C. Wu, M. Reboul, J. Y. Xiang, L. Wang, D. Lupyan, M. K. Dahlgren and J. L. Knight, *Journal of chemical theory and computation*, 2016, **12**, 281-296.
8. W. L. Jorgensen, J. Chandrasekhar, J. D. Madura, R. W. Impey and M. L. Klein, *The Journal of chemical physics*, 1983, **79**, 926-935.
9. E. Neria, S. Fischer and M. Karplus, *The Journal of chemical physics*, 1996, **105**, 1902-1921.
10. D. U. Manual, 2009.
11. G. J. Martyna, M. L. Klein and M. Tuckerman, *The Journal of chemical physics*, 1992, **97**, 2635-2643.
12. G. J. Martyna, D. J. Tobias and M. L. Klein, *The Journal of chemical physics*, 1994, **101**, 4177-4189.
